# Assessing the link between implementation fidelity and health outcomes for a trial of intensive case management by community health workers: a mixed methods study protocol

**DOI:** 10.1186/s12913-017-2320-2

**Published:** 2017-07-17

**Authors:** Barbara Schmidt, Kerrianne Watt, Robyn McDermott, Jane Mills

**Affiliations:** 1 University of South Australia, Adelaide, Australia; 2 James Cook University, Townsville, Australia; 3 James Cook University, Cairns, Australia

**Keywords:** Implementation fidelity, Primary health care, Chronic disease, Indigenous

## Abstract

**Background:**

Better systems of care are required to address chronic disease in Indigenous people to ensure they can access all their care needs. Health research has produced evidence about effective models of care and chronic disease strategies to address Indigenous health, however the transfer of research findings into routine clinical practice has proven challenging. Complex interventions, such as those related to chronic disease, have many components that are often poorly implemented and hence rarely achieve implementation fidelity. Implementation fidelity is “the degree to which programs are implemented as intended by the program developer”. Knowing if an intervention was implemented as planned is fundamental to knowing what has contributed to the success of an intervention.

**Methods:**

The aim of this study is to adapt the implementation fidelity framework developed by Keith et al. and apply it to the intervention implemented in phase 1 of the Getting Better at Chronic Care in North Queensland study. The objectives are to quantify the level of implementation fidelity achieved during phase 1 of the study, measure the association between implementation fidelity and health outcomes and to explore the features of the primary health care system that contributed to improved health outcomes. A convergent parallel mixed methods study design will be used to develop a process for assessing implementation fidelity. Information collected via a questionnaire and routine data generated during phase 1 of the study will be used to explain the context for the intervention in each site and develop an implementation fidelity score for each component of the intervention. A weighting will be applied to each component of the intervention to calculate the overall implementation score for each participating community. Statistical analysis will assess the level of association between implementation fidelity scores and health outcomes.

**Discussion:**

Health services research seeks to find solutions to social and technical problems to improve health outcomes. The development of a tool and methodology for assessing implementation fidelity in the Indigenous primary health care context will help address some of the barriers to the translation of research into practice.

**Trial registration:**

ACTRN12610000812099: 29.9.2010

## Background

Chronic disease is a major contributor to the health differentials in Indigenous populations. This is evident in Australia where the health status of Aboriginal and Torres Strait Islander people sits at the bottom of the league table of first world nations, with chronic disease the biggest contributor to burden of illness [1]. In rural and remote areas of Australia the hospitalisation rates and prevalence of risk factors of chronic disease for Aboriginal and Torres Strait Islander populations is higher than non-indigenous people [2]. It is widely recognized that better systems of care are required to address chronic disease and ensure that Aboriginal and Torres Strait Islander people have access to the care they need [3, 4].

Implementation of effective health service interventions to improve chronic disease outcomes in Aboriginal and Torres Strait Islander populations is complex. Factors that contribute to chronic illness relate to the characteristics of Indigenous populations, infrastructure in Indigenous communities and the capacity of health services to respond to Indigenous health issues. Characteristics of Indigenous populations that contribute to poor health outcomes include the social determinants of health, environmental factors and intergenerational grief and trauma caused by colonisation and racism [5]. Compared with urban areas, there is less access to comprehensive primary health care services in rural and remote locations and greater challenges with accessing specialist care [6, 7]. Workforce supply, skills and capacity to respond to health needs in a culturally competent manner, poor orientation, inappropriate service delivery models, lack of knowledge of chronic disease care guidelines, poor access to information technology and lack of resources are all barriers to effective chronic disease interventions in Indigenous Aboriginal and Torres Strait Islander populations [8–10].

Indigenous health research has produced evidence about effective models of care and chronic disease strategies that address barriers to health improvement [11–13], however the transfer of research findings from trials into routine clinical practice has proven challenging [14, 15]. Campbell et al. suggested that a solution to this problem is to strengthen research design and execution by improved theory development, modelling using qualitative and quantitative methods and evaluating long term implementation [16]. It has been suggested that poor implementation may be due to the characteristics of the intervention itself, the target setting, the research design and the interaction between these elements [15]. Another reason for poor transferability of research into practice is that research reports do not describe the intervention well enough to enable application or authentic replication in the real world setting [17]. A review of 80 studies published in the journal Evidence Based Medicine found that only 39% of non-drug trials adequately described interventions to enable replication [18]. More robust theory development and better description of interventions contribute to improved understanding of intervention components, but they do not help measure if the intervention is being implemented as intended. Therefore additional strategies are needed to monitor the implementation of interventions in a primary health care setting.

Knowing if an intervention was implemented as planned is fundamental to knowing what has contributed to the success of an intervention [19]. Complex interventions, such as those related to chronic disease, have many components that are often poorly implemented and hence rarely achieve implementation fidelity. Implementation fidelity is “the degree to which programs are implemented as intended by the program developer” [20, 21]. Breitenstein et al. state that fidelity is critical to the systematic implementation of evidence based practice [21]. Therefore health service evaluation models need to include measures that assess implementation fidelity.

The RE-AIM framework provides a model for evaluating public health initiatives by scoring the impact of an initiative in a real world setting. The dimensions measured to obtain the score include: reach, efficacy, adoption, implementation and maintenance. These dimensions are evaluated at an individual, organisational and/or community level [14]. While this model is useful for evaluating the effectiveness of chronic disease interventions in real world settings [22, 23], implementation fidelity is not being measured.

In comparison, Carroll et al. developed a conceptual model to measure implementation fidelity by allocating a score for implementation based on adherence to the intervention. The theory underpinning the model is that “implementation fidelity is the moderator between interventions and their intended outcomes” [20]. A score for implementation fidelity is arrived at by measuring adherence to an intervention which can then be correlated to health outcomes. The conceptual model provides a mechanism for assessing the effectiveness of individual components to help identify what is essential to implementing an intervention. Dimensions evaluated include: coverage, frequency and duration.

Carroll et al’s model was adapted and applied by Keith and colleagues to measure implementation fidelity of a nurse lead case management intervention for patients with cardiovascular disease [24]. Using this approach researchers and service managers were able to understand the level of implementation fidelity achieved and the impact of different components of the model on health outcomes. A key addition made by Keith et al. was the inclusion of information about context to help explain the score awarded. Implementation of any new intervention requires active change management and context plays an important role in how an intervention is received and implemented. A predictor of successful change is that the intervention proposed is consistent with the values of the organisation and there is capacity in the organisation to manage change [25]. If the innovation being proposed does not fit with the values of the organisation or is not communicated to those expected to implement it, no matter how effective the intervention may be in research conditions, it is unlikely to be implemented as planned. Measuring implementation fidelity and analysing results using a theoretical lens of change management would assist managers to improve service planning for complex chronic disease interventions and better manage the risks with implementation of chronic disease interventions in Aboriginal and Torres Strait Islander communities.

## Methods/design

The aim of this study is to adapt the implementation fidelity framework developed by Keith et al. and apply it to the communities that participated in phase 1 of the Getting Better at Chronic Care in North Queensland (GBACC) study. The GBACC study was a cluster randomised control trial (RCT)of intensive case management by Indigenous Health Workers of people with complex chronic disease care needs [26]. The primary outcome measure was reduction in HbA1c over 18 month and there were a range of secondary measures including improvement in clinical process measures and hospital avoidance for ambulatory sensitive chronic conditions. The study produced modest results and a possible reason for this is implementation failure of the intervention. This study will quantify the level of implementation fidelity achieved during phase 1 of the GBACC study and investigate the features of the primary health care system that contributed to successful improvement in clinical measures and hospital avoidance for ambulatory sensitive conditions.

The objectives of the study are to:

1. Investigate the features of primary health care service models that contribute to intervention effectiveness for chronic disease care of Indigenous patients and identify if they are present in the context of health services participating in the GBACC study.
2. Assess the level of implementation fidelity achieved for each component of the intervention in each community.
3. Measure the level of implementation fidelity achieved in each community
4. Determine if there is any association between implementation fidelity and health outcomes as measured by HbA1c and preventable hospitalisation for diabetes related conditions for communities in the GBACC study.

The study has approval from the Far North Queensland Human Resource Ethics Committee (HREC), University of Queensland HREC and the University of South Australia HREC.

**Fig. 1:**
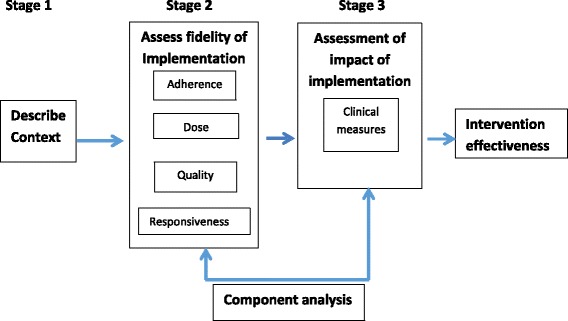
Model for assessing Implementation fidelity of GBACC model. *(Adapted from Carroll (2007) and Keith (2010))*

A convergent parallel mixed methods design will be used to develop the measures for assessing implementation fidelity for each component of the intervention implemented during phase 1 of the study. The study will be executed in three stages using a model adapted from Carrol (2007 and Keith (2010). The analysis will use routine data generated by the GBACC study, clinical data collected to measure the outcome from the RCT and additional information collected via a questionnaire to describe the health service context in which the study occurred. The model is illustrated in Fig. 1.

### Stage 1 – Development of tools to describe context and measure implementation fidelity

The stage 1 objective is to develop tools to collect information that explains the service delivery in the intervention sites and confirm the measures to be used to assess implementation fidelity.

#### Development of measures for implementation fidelity

The GBACC model of care (the intervention), the theory underpinning the model and each component of the model was documented during phase 3 of the GBACC study. The description of the model and the measures for assessing each component and the data sources proposed to assess implementation of each component were discussed with stakeholders at a workshop in May 2015. Modifications were made to the description of the component and consensus achieved on measures to be used to assess implementation fidelity of the GBACC study.

#### Describing the context for primary health care service delivery

A systematic literature review will be completed to identify the features of health services for Indigenous communities that contribute to effective primary health care interventions. The results will be used to develop a questionnaire to collect information about the health service context in the six intervention sites of the GBACC study. The objective of the questionnaire is to understand the management and service delivery support structures in place to support the intervention.

### Stage 2 – Assessment of implementation fidelity

The Stage 2 objective is to collect and analyse information about the service context and implementation of the model components. Information about model components will be used to calculate implementation fidelity scores for each component. Service context information will be analysed to identify enablers and barriers to implementation of components.

#### Service context questionnaire

The service context questionnaire, stakeholder information sheet and a consent form will be emailed to the Primary Health Care Manager or equivalent in the intervention sites (*n* = 6) to complete. A follow up interview will be conducted the health service managers to explore any missing information and confirm the responses to the questionnaire. The completed questionnaires will be transcribed, collated and coded using nVivo.

#### Scoring model components

**Fig. 2:**
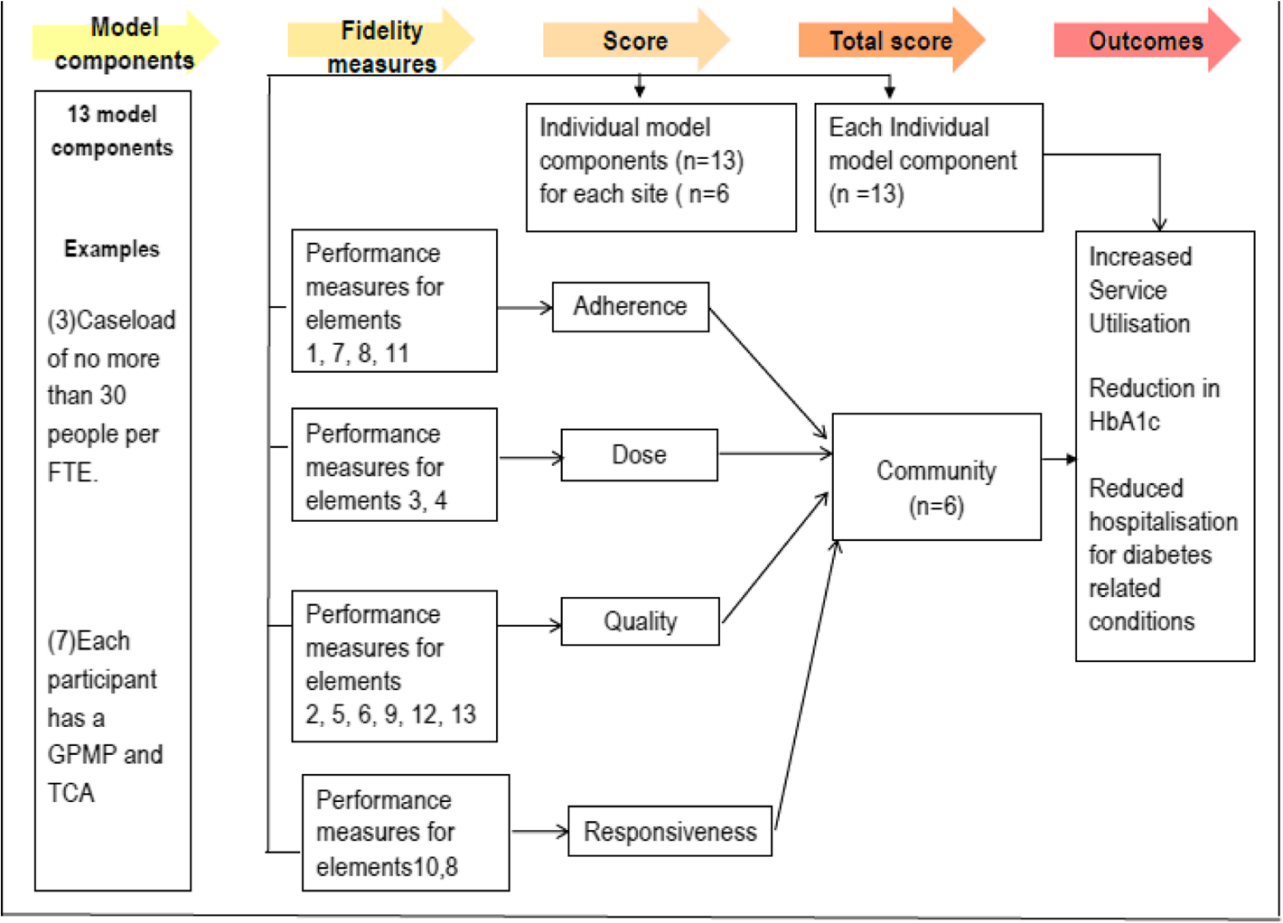
Directed Acyclic Graph (DAG) - Implementation fidelity GBACC project

A directed acyclic graph (Fig. 2) illustrates the measures proposed to assess implementation fidelity and map to the dimensions of adherence, dose, quality, and responsiveness. Some measures will be dichotomous (i.e yes or no) while others will be assigned a percentage score based on the level of implementation that occurred. Per protocol analysis will be followed when determining compliance. A weighting for each element will be assigned to reflect the importance of the element. It is expected that additional criteria will need to be developed to define compliance. A workshop will be held with key stakeholders to validate the scoring process and agree on weightings that should be applied to different model components, for each dimension.

A series of excel tables will be generated that shows the raw data and implementation score assigned for each component. All model components will be aggregated by community to develop an implementation fidelity score for each community.

### Stage 3: Assessment of intervention effectiveness

The objective of stage 3 is to assess the impact of the program by looking at the association between the implementation fidelity score and outcome effectiveness. The aim is to determine if it is possible to predict outcome effectiveness based on the features of the PHC system. Intervention effectiveness (i.e implementation fidelity score for each component) is the independent variable and avoidable hospitalisations and service utilisation is the dependent variable.

**Fig. 3:**
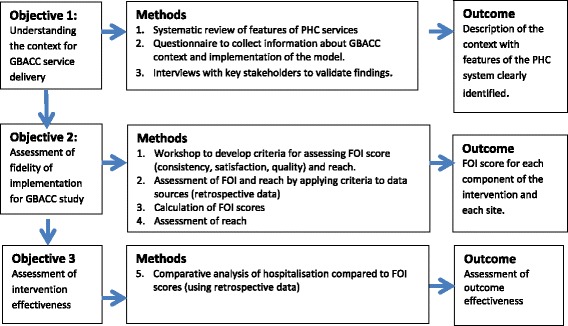
Study design Implementation fidelity of GBACC study

Figure 3 provides a summary of the study design to assess implementation fidelity of phase 1 of the GBACC study.

## Discussion

Health services research seeks to find solutions to social and technical problems to improve health outcomes. Health services research is more complex than scientific research alone because it deals with societal problems in health care. In the Indigenous health setting the problems being addressed are often considered ‘wicked problems. That is the setting is complex, and there are many different views on the causes of poor indigenous health (the problem), the mission to solve problems is not always clear and there is not one agreed solution to the problem [27]. RCTs that use a mixed methods design provide more evidence and insight into the ‘societal’ issues to explain the outcomes achieved from RCT. However gaps often remain in our understanding of the scope of implementation that occurred and the impact that different components of an intervention had on outcomes. The development of a methodology for assessing implementation fidelity in the context of Indigenous primary health care health setting will help address some of the barriers identified with the translation of research into practice [15, 28] and produce better evidence about factors contributing or preventing successful implementation of programs in the research and real world setting.

## Abbreviations

GBACC: Getting better at chronic care in North Queensland study
HREC: Human Research Ethics Committee
RCT: Randomised control trial.

## References

[1] Oxfam. Closing the Gap: Solutions to Indigenous Health crisis facing Australia. Fitzroy: Oxfam; 2007.

[2] Australian Institute of Health and Welfare (2014). Australia Health 2014, in Australia’s health.

[3] Wagner E (2001). Improving chronic illness care: Translating evidence into action. Health Aff.

[4] Liddy C (2013). The Community Connection Model: Implementation of best evidence into practice for self-management of chronic diseases. Public Health.

[5] Griew R. The link between primary health care and health outcomes for Aboriginal and Torres Strait Islander Australians. Canberra: Robert Griew Consulting; 2008.

[6] Phillips A (2009). Health status differentials across rural and remote Australia. Aust J Rural Health.

[7] Carey T (2013). What primary health care services should residents of rural and remote Australia be able to access? A systematic review of "core" primary health care services. BMC Health Serv Res.

[8] Wakerman J (2008). Primary health care delivery models in rural and remote Australia - a systematic review. BMC Health Serv Res.

[9] Gibson O (2015). Enablers and barriers to the implementation of primary health care interventions for Indigenous people with chronic diseases: a systematic review. Implement Sci.

[10] Schmidt B, Campbell S, McDermott R (2015). Community health workers as chronic care coordinators: evaluation of an Australian Indigenous primary health care program. Aust N Z J Public Health.

[11] Knight A (2012). Improving primary care in Australia through the Australian Primary Care Collaboratives Program: a quality improvement report. BMJ Qual Saf.

[12] Wise M. National Appraisal of Continuous Quality Improvement Initiatives in Aboriginal and Torres Strait Islander Primary Health Care: Final report. Melbourne: Lowitja Insititute; 2013.

[13] Schierhout G (2013). Evaluating the effectiveness of a multifaceted, multilevel continuous quality improvement program in primary health care: Developing a realist theory of change. Implementation Sci..

[14] Glasgow R, Vogt T, Boles S (1999). Evaluating the public health impact of health promotion interventions: The RE-AIM framework. Am J Public Health.

[15] Glasgow RE, Emmons KM (2007). How Can We Increase Translation of Research into Practice? Types of Evidence Needed. Annu Rev Public Health.

[16] Campbell M (2000). Framework for design and evaluation of complex interventions to improve health. BMJ.

[17] Glasziou P (2010). Taking healthcare interventions from trial to practice. BMJ..

[18] Glasziou P (2008). What is missing from descriptions of treatment in trials and reviews?. Br Med J.

[19] Damschroder L (2009). Fostering implementation of health services research findings into practice: a consolidated framework for advancing implementation science. Implement Sci.

[20] Carroll C (2007). A conceptual framework for implementation fidelity. Implement Sci.

[21] Breitenstein SGD, Garvey C, Hill C, Fogg L, Resnick B (2010). Implementation Fidelity in Community - Based Interventions. Natl Insititute Health.

[22] Glasgow R (2001). The RE-AIM framework for evaluating interventions: what can it tell us about approaches to chronic illness management?. Patient Educ Couns.

[23] Glasgow RE (2006). Using RE-AIM Metrics to Evaluate Diabetes Self-Management Support Interventions. Am J Prev Med.

[24] Keith R (2010). Fidelity of implementation: development and testing of a measure. Implement Sc.

[25] Klein K, Sorra J (1996). The Challenge of Innovation Implementation. Acad Manag Rev.

[26] Schmidt B (2012). Getting better at chronic care in remote communities: study protocol for a pragmatic cluster randomised controlled of community based management. BMC Public Health.

[27] Rittel H, Webber M (1973). Dilemmas in General Theory of Planning. Policy Sci.

[28] Kessler R, Glasgow R (2011). A proposal to speed translation of healthcare research into practice: dramatic change is needed. Am J Prev Med.

